# A critical narrative review of the experiences of Latinas, Hispanic, or Spanish origin (LHS+) women in medical field using Latina/Chicana feminist perspective

**DOI:** 10.1080/10872981.2025.2534049

**Published:** 2025-07-17

**Authors:** Sacha Sharp, Ashley Hixson Clarke, Berenice Sánchez, Julia C. Stumpff, Sylk Sotto-Santiago

**Affiliations:** a Department of Medicine, Indiana University School of Medicine, Indiana, IN, USA; b Equity, Inclusion, Belonging, Center on Budget and Policy Priorities, Washington, DC, USA; c School of Psychology and Educational Leadership, Idaho State University, Pocatello, ID, USA; d School of Medicine, Indiana University, Indianapolis, IN, USA; e Department of Medicine, University of Pittsburgh, Pittsburgh, PA, USA

**Keywords:** Latinas, Hispanic or Spanish origin, women, identity, medical education, narrative review, ethnic representation

## Abstract

Women who identify as Latinas, Hispanic, or Spanish Origin (LHS+) are members of one of the fastest growing ethnic groups in the US. When we consider this fact regarding providing quality healthcare, we find that it is important to have a physician workforce that is representative of this population. However, there is little research about the experiences of LHS+ women in the medical field. The purpose of this study was to explore narratives found in medical education literature documenting the experiences of LHS+ women as learners, trainees, and professionals across the medicine continuum. We conducted a critical narrative review using Latina Chicana Feminist Perspective as a theoretical framework to identify and synthesize literature on LHS+ women in medicine. From July 2021 to October 2021 and November 2023 to January 2024, we performed searches using one database (MEDLINE [OVID]) and selected studies consistent with narrative integrity and relevance to the experiences of LHS+ women using the data software Covidence. After four phases of review, which included the identification phase, screening phase, and eligibility phase, we found 12 articles that discuss the experiences of LHS+ women. The literature allowed us to provide a preview of what is being discussed in terms of the experiences of LHS+ women populations. Although the articles found provided some information about the experiences of LHS+ women in medical education literature, more information is needed. Given the limited representation of narratives about LHS+ women in medical education research, there is a narrow opportunity to explore what their medical school experiences are and have been to develop interventions for their success. Medical educators and administrators are therefore limited in how they can address the possibilities of enhancing or replicating positive experiences and environments for LHS+ women in medicine.

## Introduction

As members of one of the fastest-growing ethnic groups in the United States, the medical education and training of individuals who identify as Latina/o/e, Hispanic, or Spanish Origin+ (LHS+) [1] is important as we consider their future contribution to the physician workforce [2]. Members of the LHS+ community are more likely to be bilingual and tend to be younger, with a median age of 30.5, factors necessary for serving the healthcare needs of a growing multilinguistic and aging population in the United States. Researchers also suggest that the educational advancement of LHS+ populations is proven to have positive impacts on the socioeconomic stability of the United States [3]. This could be attributed to LHS+ population growth, with members making up 19.5% of the population [4]. Consequently, there is limited medical education literature that discusses the educational needs of members of the LHS+ community, especially women.

According to the Association of American Medical Colleges, and organization most trusted for medical education data in the United States [5], LHS+ women comprise only 3.5% of the medical school enrollment population. The term, Latina/o/e, Hispanic, or Spanish Origin+ or LHS+ refers to the various ethnic categories that include Spanish heritage such as Hispanic and Chicano [1]. Concerning the physician workforce, LHS+ women only comprise 2.4% of the physician population [6]. These numbers are inconsistent with the representation of LHS+ women in society, who made up around 9.6% of the population in 2023 [4]. This is an issue given that patients disproportionately seek out concordant racial, gender, and linguistic physician relationships [7]. Researchers also suggested that increased diversity in the physician workforce contributes to positive patient health outcomes and can help to improve health equity [8,9]. There is great concern for the recruitment and retention of LHS+ women into medical schools and the medical physician workforce for the purpose of representativeness. However, there is little information about the retention of LHS+ women in these medical education environments. Because ways to promote retention are often connected to how individuals experience the medical education environment, we find it necessary to fill the gap in the literature that discusses the experiences of LHS+ women in medicine in hopes of determining why they may stay or leave the profession. Therefore, the purpose of this study was to perform a critical narrative review of the literature about LHS+ women to uncover how they experience, survive, and thrive as learners, trainees, and professionals across the medicine continuum. Our critical narrative review is guided by the following question:

What does the medical education literature explicitly say about the experiences of LHS+ women professionals across the medical continuum (medical school, residency, fellowship, and physicians)?

## Literature review

Medical education researchers highlight the importance of diversity, equity, and inclusion (DEI) [10,11]. However, both students and faculty referred to as underrepresented in medicine (URIM) still experience racial and gender discrimination in academic medicine [12,13]. This discrimination exists in the form of microaggressions, harassment in the form of ‘pimping,’ limited access to leadership roles because of institutional barriers, and systemic racism. The existing literature on URIMs also reports aggregated data on minoritized groups. Therefore, medical education literature fails to capture the nuanced and unique experiences of URIM individuals with multiple marginalized identities, such as LHS+ women. For example, because the data on experiences with harassment is often aggregated for all URiMs, we cannot decipher the types of unique experiences LHS+ women may have when compared to LHS+ men. Moreover, we cannot assess the nuanced experiences had by LHS+ individuals when compared to their Black individual counterparts. As noted, research that exclusively focuses on LHS+ women is scarce. For this literature review, we draw from higher education literature that specifically focuses on LHS+ women to help illuminate the necessity for taking a decolonial approach that strives to center the voices of marginalized communities and dismantle hegemonic power structures to research about this minoritized group in medicine.

Despite the increasing number of LHS+ women entering the academy as graduate students and faculty, they continue to experience discrimination and dissuasion [14]. Researchers have found that LHS+ women graduate students tend to have fewer professional socialization opportunities compared to their men and white women counterparts [15]. In the classroom setting, women LHS+ students experience microaggressions that may negatively impact their self-perception and intellectual ability for academic success [2]. Furthermore, LHS+ women are regularly one of a few if not the only women of color within their graduate or professional programs, which leads to a sense of marginalization, invisibility, and tokenization as they are left to navigate their program on their own if they are not able to find a faculty or peer to serve as a mentor or source of support [16].

The experiences of marginalization and discrimination are ongoing for LHS+ women once they enter the professoriate. LHS+ women faculty report that they are not perceived as credible or legitimate scholars and researchers among their peers, which ultimately devalues their contributions to the academy [17]. This devaluation leads many LHS+ women faculty to doubt their own competence, especially within fields where they are underrepresented like the STEM disciplines [18]. As a result of all the challenges that LHS+ women face within the professoriate, they are often left to find or create sources of support to successfully navigate the academy [19]. Ultimately, LHS+ women must demonstrate resilience in maneuvering predominantly white, male spaces in higher education.

Despite the many challenges that LHS+ women face within academia; they turn to various sources of support as they successfully navigate their educational and professional journey. One major source of support comes from the peer support networks they form for themselves. These peer networks are often established during their graduate programs and continue to develop once they enter their professional roles [20]. The support of family also has a major positive impact on LHS+ women in academia. Familial support appears in the form of immediate and extended family, as well as community members who are there for the women when issues arise. These individuals and community members serve as motivation to encourage the women through ups and downs [21]. Lastly, research suggests that LHS+ women turn to personal agency and resilience to navigate the academy. LHS+ women’s resilience throughout their educational journey regularly centers on home, school, college, community, and work as different contexts that shape their experiences, growth, and successes within higher education [22,23].

The educational experiences LHS+ women have as students and faculty outside of medical education may be translated to those experiences in medical education, where the overall demographics and underrepresentation are consistent. Existing literature provides some insight, but more research is needed to account for the educational context within academic medicine. Thus, we conducted a critical narrative review using the Latina Chicana Feminist perspective to expand further the literature on the experiences of LHS+ women in medical education.

## Researcher positionality

Before sharing more about the Latina Chicana Feminist perspective (LCFP), which serves as our theoretical framework, we believe it is important to offer our positionality as researchers. The research team is comprised of two Black American women, two American women of Latina/Chicana descent, and one white American woman. One of the Black women researchers is a faculty member in academic medicine, and one is a PhD administrator for a non-profit organization. The first Chicana woman is a faculty member in higher education, while the other is a tenured Latina faculty member in academic medicine. The white woman is a medical librarian and faculty member in academic medicine. This research is significant to each member of the research team because we regularly interact with graduate and professional students, and all but the white woman sees ourselves represented in the demographic being examined for this critical narrative review. Specifically, the women of color in the research team have experienced first-hand the lived intersectional experience of being a woman of color and dealing with racism and sexism within academia both in and outside of medicine. Furthermore, each member of the team understands the significance of centering LHS+ women in the medical education discourse, as doing so will unearth and hopefully eliminate the potential barriers these women experience, thus leading to academic persistence and success for this demographic.

## Theoretical framework

**Figure 1.**
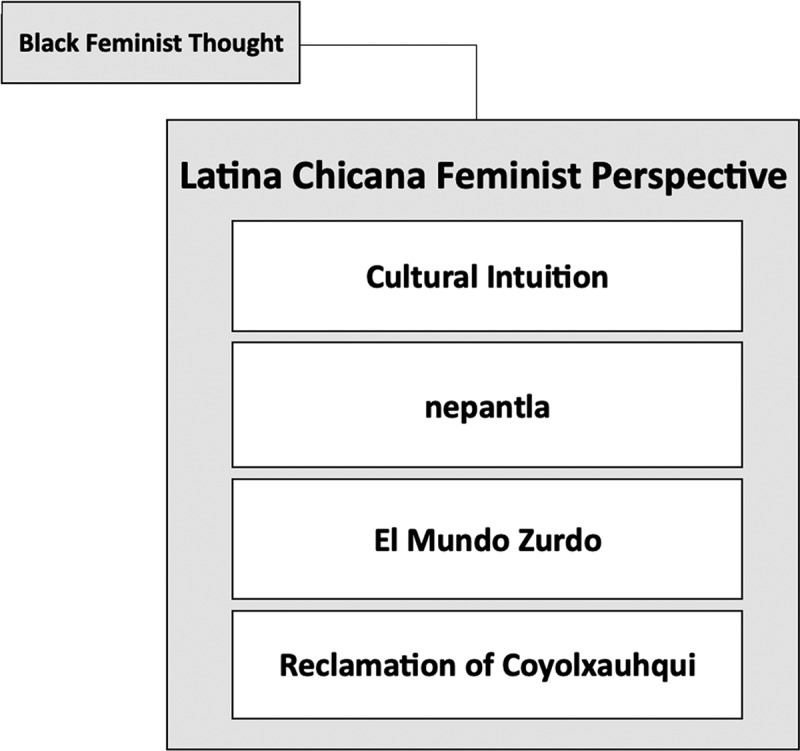
Model of Latina Chicana feminist perspective.

We used Latina Chicana Feminist perspective (LCFP) as the theoretical and analytical framework for this critical narrative review (Figure 1). Informed by aspects of Collins’ [24] Black feminist thought (BFT), LCFP centers the experiences of LHS+ women in the creation and understanding of knowledge [25]. Within education research, Calderón et al. [26], situated the use of LCFP ‘as a response to the failure of both mainstream education research and liberal feminist scholarship to address the forms of knowledge and experiences Latinas/Chicanas bring to educational institutions and research’ (p. 515). A central aspect of LCFP is the idea of cultural intuition, which highlights the unique perspective that LHSs+ women bring to the knowledge-creation process. This concept is like the outsider-within perspective that Black women hold as discussed in BFT [24]. Cultural intuition draws from LHS+ women’s unique personal and professional experiences with navigating multiple systems of oppression, existing literature on a topic, and the analytical research process used to create knowledge [25]. The use of cultural intuition allows for the recognition of LHS+ women’s diverse perspectives and experiences, but acknowledges that LHS+ women are not a monolith.

In conjunction with our cultural intuition, we also utilized theoretical concepts offered by Anzaldúa [27] that are often associated with LCFP. Anzaldua proposed concepts like nepantla, El Mundo Zurdo, and reclamation of Coyolxauhqui [27]. nepantla refers to the idea of living between worlds and having multiple beliefs. For scholars to evoke nepantla means to have an appreciation for the various knowledge sources that exist outside the binary thinking that maintains white supremacy [28]. The theme of nepantla explains how scholars, particularly LHS+ women scholars, disrupt power and the various contradictions that can often lead to social injustice. El Mundo Zurdo is another theme offered by Anzaldúa [27], which considers the queering of space, body, and place found at the margins to expose oppression and decolonize educational practices. Using El Mundo Zurdo, scholars embrace ambiguity and promote healing and liberatory practices. Finally, the concept of the reclamation of Coyolxauhqui [27] highlights the process of healing with words that reconnect the mind, body, and spirit. As a research team consisting of all women, and majority women of color, we pulled from the broader aspects of cultural intuition (i.e., personal and professional experiences, existing literature, and analytical research process) and the concepts of Nepantla, El Mundo Zurdo, and Coyolxauhqui as the lens through which we review the manuscripts uncovered through the critical narrative review process. The use of a Latina Chicana Feminist Perspective allowed the research team to explore the nuanced experiences of LHS+ women as presented in the literature. More details regarding our analytical approach are shared in the sections that follow.

## Methods

We conducted a critical narrative review [29] where we performed a methodical search to identify and synthesize medical education literature about the experiences of LHS+ women. That approach consisted of a) the search was conducted only using one database (MEDLINE (OVID)), b) only three members of the team collaborated to make study inclusion and coding decisions, and each member worked independently before comparing results, c) the studies included in this review were selected based on narrative integrity or narratives that are consistent, accurate, and avoid misleading information, and relevance to the experiences of LHS+ women, not methodological quality, d) the process did not follow methods used for a systematic review. Because this review was designed to focus on the experiences of LHS+ women in academic medicine, we were not concerned with the possible limitations of critical narrative review that often include selection bias or opinion articles. Consistently, we included perspectives as these articles provided insights into the experiences of LHS+ women we would not have otherwise.

### Search strategy

Using a variation of search terms (e.g., ‘Hispanic,’ or ‘Latina,’ and ‘Latinx,’ and ‘women’ and ‘medical student’ and ‘resident,’ or ‘trainee’) we performed a broad electronic database search through the National Library of Medicine (NLM) principal biomedical bibliographic broad electronic database (MEDLINE [OVID]). We chose this database because it houses most of the articles written about academic medicine in the US. The initial search was conducted on 7 July 2021 and took 2 days to complete, which included discussing the appropriate search terms. These data were then uploaded to data software Covidence where the narrative review process was conducted, which lasted until October 2021. This initial review only yielded three articles. After much discussion and feedback from scholars in the field, we elected to perform an additional search process. Moreover, significant changes in the US political landscape such as the SFFA v. Harvard/UNC decision regarding the elimination affirmative action made it so that more literature on the topic of LHS+ women’s experiences could be yielded. The initial search for this second phase was conducted on 30 November 2023 and took less time to complete given it was a replication of the previous search. The complete narrative review process lasted through January 2024. Manuscripts that included the following criteria were selected for analysis:

- explicitly discussed experiences of Latinas or women of Spanish descent as learners or faculty in a medical context
- were published with an American publishing company
- available in full-text
- published in English
- dates ranging from open through day search conducted

Because our research question asks what the medical education literature explicitly says about the experiences of LHS+ women, our first criteria involved the inclusion of manuscripts that specifically discuss experiences of LHS+ women in the medical field. We sought out articles published with American companies because we wanted to find materials that were congruent with an American context for how medical education is delivered. To read the complete articles, we found only manuscripts in full text. Because the research team mostly speaks English, and because many manuscripts were published in English, we included materials written in English. This also allows readers to engage with the manuscripts found in this review since English is a common language among many. Finally, the date ranges for the articles found in this critical narrative review were open because we wanted to find as many manuscripts as possible. Articles that did not meet these criteria were excluded from the study. The full search strategy included the following terms:

- ‘medical school(s)’
- ‘medical student(s)’
- ‘Internship and residency’
- ‘learner or trainee or resident’
- ‘faculty’
- ‘Clinical clerkship or education’
- ‘women or woman or female’
- ‘Hispanic or Latina/o or Spanish Descent’

### Delimitations

Because our inclusion criteria consist of only using articles offered as full text, published in English, and published in the United States, this review is not considered an exhaustive list of the literature that may be available on the experiences of LHS+ women in academic medicine. However, our approach is consistent with the requirements of a critical narrative review. Moreover, the articles found were those published until November 2023; therefore, we may have missed manuscripts published after this date.

### Procedure

**Figure 2.**
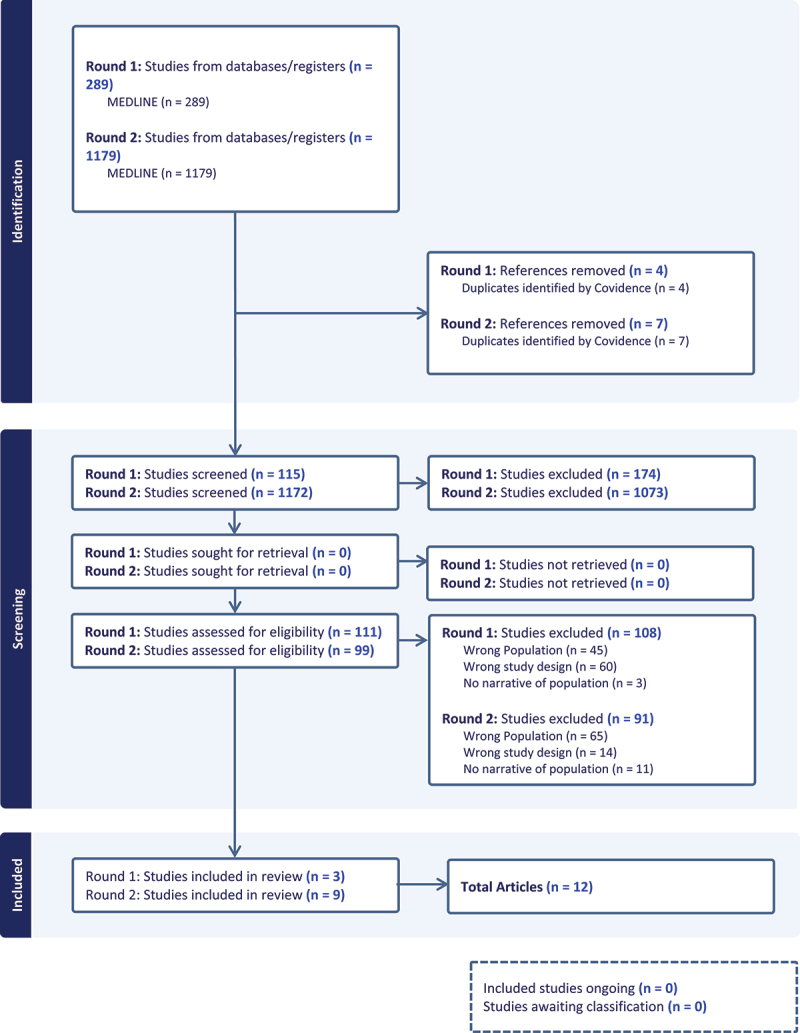
Diagram of the search and selection process administered July 2021 and November 2023.

Data sources were selected in four phases (Figure 2). In the first phase called the identification phase, two authors worked to methodically search the MEDLINE (OVID) database for articles based on the search terms referenced above. This search procedure occurred in two rounds with round one taking place in 2021 and yielding 289 articles, and round two occurring in 2023 and yielding 1172 articles. During phase two or the screening phase, three research team members reviewed all manuscript titles and abstracts to determine relevance. Of the 289 articles in the first round, we determined only 115 articles to be potentially relevant. Articles evaluated in the second round yielded 99 potentially relevant articles based on the abstracts. We designated these articles as potentially relevant in this initial review phase because many of the articles referenced both women and LHS+ populations in the abstract but did not explicitly mention LHS+ women. Therefore, we felt it was necessary to scan the articles to determine if LHS+ women were being discussed.

In the third phase or the eligibility phase, we conducted a brief scan of the articles to determine whether LHS+ women were explicitly mentioned in the narrative or discussion of the manuscript. Of the manuscripts in the first round, we found only three articles met the inclusion criteria. Of the articles assessed in the second round, we determined that 19 articles mentioned LHS+ women. Finally, we did a deep read of the manuscripts to determine the context of how LHS+ women were being discussed. In the first round, we found that all three articles did mention LHS+ women in terms of being faculty physicians and therefore met the inclusion criteria. However, none of those manuscripts highlighted the experiences of LHS+ women medical students or residents. Moreover, only one of the three articles centered on LHS+ women as the population for the study. For round two, we found 9 of the 19 to meet the criteria. Ten of the original 19 articles referenced LHS+ populations but did not explicitly discuss LHS+ women. When LHS+ women were mentioned in those articles, they were referred to as patients or members of a different health profession from medicine. We were left with a total of 12 articles to review in our analysis.

## Analysis

In our analysis of the 12 manuscripts (Table 1), we utilized our cultural intuition along with nepantla, El Mundo Zurdo, and the reclamation of Coyolxauhqui to guide our deductive analysis process. Our personal and professional experiences as women and people of color in the academy and knowledge of the broader body of literature on LHS+ women in higher education directed how we incorporated Anzladúa’s [27] concepts as we analyzed and interpreted the narratives found in these manuscripts. We used nepantla by exploring whether the articles centered on the unique experiences of LHS+ women as they navigated medical education and acknowledged the knowledge offered by these women. We were especially interested in manuscripts that exposed power imbalances, conflict, and contraindications that privileged Eurocentric approaches to research. In seeking this type of information, we were comparing what we know from other fields, such as higher education, to that of medical education.

**Table 1.** Characteristics of eligible studies.

Study ID	Title	Country in which study conducted	Aim of study	Study design	Population description	Narative about population
Capdevila-Gaudens 2021	Depression, anxiety, burnout and empathy among Spanish medical students.	Spain	To conduct a nationwide analysis of the prevalence of mental health problems among medical students.	Cross sectional study	Of the 5,198 respondants from 43 medical schools in Spain, 76.3% (3979) were women.	In relation to depression, the data indicate an overall prevalence of 41%, with 23.4% of participants having moderate to severe levels, and 10% experiencing suicidal ideation. Burnout prevalence was 37%, significantly higher among 6th year than among 1st year students. Anxiety levels were consistent with those reported previously among medical students (25%), and were higher than in the general population for both trait and state anxiety. The prevalence of trait anxiety was higher among women. Empathy scores were at the top end of the scale, with the highest-scoring group ( > 130) containing a greater percentage of women.
Gerk 2022	Gender Discrimination, Career Aspirations, and Access to Mentorship Among Medical Students in Brazil.	Brazil	To investigate the impacts of Gender Based Discrimination (GBD) on career selection and well-being of medical students in Brazil and to explore access to mentorship among these students	Cross sectional study	Of 953 respondents, 748 (78%) were cisgender women.	In Brazil, the medical school training lasts 6 y (12 semesters),with the two final years dedicated to clinical clerkship rotations. We included students enrolled in any academic year from a Brazilian medical school recognized by the Ministry of Education. Participants were obtained through convenience sampling. Participation was voluntary, and no compensation was offered. Using a survey sample size calculation with a 5% margin of error and 95% confidence interval, we estimated the need for approximately 370 respondents
Faria 2023	Gender-Based Discrimination Among Medical Students: A Cross-Sectional Study in Brazil.	Brazil	To explore the patterns of gender-based discrimination (GBD) experienced by medical students in Brazil	Cross sectional study	Of the 953 respondants from all medical schools in Brazil, 748 were women.	Women were more likely to experience and witness GBD than men. Women experienced GBD from professors, specialist medical doctors, circulating nurses, and relatives of patients. They were more likely to be discriminated against by men faculty, specialist medical doctors, and students at all years. Women were more likely to experience discrimination on their medical school campus, in the classroom, inpatient wards, operating room, and preoperative area. Women were more likely to report not knowing about a reporting mechanism or knowing if there was one. In 2020, women represented 59% of medical students and 46% of physicians in Brazil.
Juanico-Morales 2023	Depression and associated factors in medical students in Acapulco during the COVID-19 pandemic: A cross-sectional study.	Mexico	To assess depression prevalence and its associated factors affecting medical students in Acapulco, Mexico during the COVID-19 pandemic	Cross sectional study	Of the1288 participants included in the analysis, 63.4% (817/1288) of respondents were women.	The survey included 95% (1416/1486) of those regularly attending classes. The remaining 70 declined to participate and we have no details on how they differed from the respondents. We excluded 123 respondents who reported a diagnosis of depression before entering university and five potential participants aged under 18 years. The analysis thus included 1288 students (Fig 1) of whom 52.6% (677/1288) in their first four semesters of the eight-semester plan of studies. The mean age was 21 ± 2.3 years; 63.4% (817/1288) of respondents were women. 19.6% (252/1288) reported a family history of depression. Table 1 shows participant characteristics.
Sanchez-Medina 2023	Making mentoring more impactful for URiM students.	USA	To offer perspective from a Latina lead author who is expressing a model for URiM mentorship.	Perspective	This paper highlights the authors perspective.	In her experience, the impact of formal mentoring as a Latina medical student was significant.
Balderas-MedinaAnaya 2022	Latina Women in the U.S. Physician Workforce: Opportunities in the Pursuit of Health Equity.	USA	To examine progress made toward Latina representation in the physician workforce with an emphasis on health equity. Filling the gap of research about Latina physicians.	Cross sectional study	Latinas make up 2.4% if the total U.S. physician population.	Hispanic women tend to be youngest with the median age of 40 and only few hispanic women being 65. 68.8% of hispanic women speak Spanish at home. 59.9% of hispanic women physicians are US born and 40.1% are immigrants. Hispanic women physicians are more than twice as likely to be naturalized citizens. The authors offer suggested for workforce recruitment based on the demographic trends of hispanic women. Latinas over 33 times more likely to speak Spanish, and therefore should be recruited to balance language concordance in the physician workforce for purposes of improving physician outcomes. Cultural concordance is also important for avoiding implicit bias. Cultural concordance can mitigate stereotypes that prevent misdiagnosis and healthcare inequity. Research on Latinas is also important to include in clinical trials. Future research could include why Latinas are under enrolled in medical school. Why aren’t diversity efforts in medical school successful for Latinas? What is the structural barrier in the education pipeline for Latinas and other Hispanic students? Only 3.2% of medical school faculty are hispanic.
Kamran 2022	Intersectional Analysis of U.S. Medical Faculty Diversity over Four Decades.	USA	To explore whether the clinical faculty and leadership representation at academic medical schools reflect the diversifying population between 1977 and 2019	Quantitative secondary data	NA, numbers not disagregated for Latinas.	The representation of Latinas as clinical faculty has surpassed Black women over time, yet they remain underrepresented.
Saxena 2023	Trends of Academic Faculty Identifying as Hispanic at US Medical Schools, 1990–2021.	USA	To examine number of full-time US medical school faculty who identify as Hispanic.	Cross sectional study	NA	Lag between Latinas and Latinos in medicine. Increases in faculty do not match population increases for Latina population. 0.76% in 1990 and 2.74% in 2021 for Latinas. Women largely assistant professors. Suggestions are made for how to increase representation of latine students.
Lugo-Fagundo 2023	From medical school through residency applications: Puerto Ricans may face ignorance and prejudice.	USA	To promote awareness of potential biases at different stages of medical education	Qualitative research	The study includes the experiencs of two Puerto Rican women physicians.	Participant 1: This physician was born and raised in Puerto Rico, completing her primary education on the island. She later moved to the contiguous U.S. where she completed her undergraduate and medical school degrees. Currently, she is a second-year dermatology resident at an institution on the U.S. mainland. Participant 4: She is a resident who was born in the contiguous U.S. before her family moved to Puerto Rico. Upon completion of her primary education on the island, she moved to the mainland U.S. where she completed her undergraduate degree before returning to Puerto Rico for medical school. She is currently a second-year interventional radiology resident at an institution in the continental U.S.
Lopez Leon, 2019	Medical careers and motherhood: A cross-sectional study of Hispanic female physicians	USA	To explore the speciality choice, childbearing, and professional and personal life characteristics, along with respondents’ suggestions for Hispanic female physicians who want to start a family, and how hospitals and medical institutions ocould engance their support of female medical staff members with children.	Cross sectional study	This study includes 1,241 Hispanic female physicians who were mothers.	Most respondants (83%) did not take off significant time to care for the new baby. The specialties that are often selected by Hispanic women due to the ability to control or balance lifestyle – specialties being primary care oriented or generalist. Common suggestions for institutions to engance support of female physicians related to providing childcare, lactation facilities, and space for families who accompanied female physicians for brief visits to attend to patient needs. The women also suggested more flixibility in work and vacation schedules. Forty-sevel percent of respondents had their first child during medical school or residency, which are the times of high work demands.
Jones, 2015	Racial and ethnic disparities in cardiovascular disease: An assessment of obstetrician-gynecologists’ knowledge, attitudes, and practice patterns	USA	To assess whether OB/GYN race/ethnicity and OB/GYN practices with increasing minority patient populations predicted differences in OB/GYN’s knowledge, attitudes, and practice patterns relevant to racial/ethnic disparities in CVD.	Quantitative study	ACOG fellows and junior fellows who are active members of the Collaborative Ambulatory Research Network (CARN)	Women of Spanish descent OBGYN physicians were more knowledgeable about the risks of cardiovascular disease for women of the same ethnic group than their white women counterparts.
Mahoney, 2008	Minority faculty voices on diversity in academic medicine: Perspectives form one school	USA	To examine the perceptions and experiences of ethnic minority faculty at UCF regarding racial and ethnic diversity in academic medicine, in light of a constitutional measure outlawing race- and gender-based affirmative action programs by public universities in California.	Qualitative research	Faculty in a database maintained by the Underrepresented in Medicine Mentorship Program. 25 interviews conducted (16 were Latino)	Quotes from a senior rank Latina faculty member were highlighted throughout the manuscript, and the findings included experiences of discrimination, lack of implementation around increasing diversity, and limited mentorship options.

The concept of El Mundo Zurdo allowed us to consider whether and how the relevant manuscripts reviewed portrayed research that would make sense of LHS+ women experiences and lead to a transformation of current practices that negatively impact those experiences. Additionally, we sought out how scholars discussed personal healing as tied to the Earth. Lastly, we were interested in Coyolxauhqui or whether the manuscripts found as part of the critical narrative review were able to honor LHS+ women by holistically recognizing them rather than separating their minds, bodies, and spirits from the text. We paid particular attention to researchers who highlighted nepantla or the various intersecting identities and roles that LHS+ women carry with them and acknowledged that being in medicine was just one of those identities for these women.

## Results

For the presentation of results, we first provide an overview of what the 12 articles highlighted about the experiences of LHS+ women. While not all 12 articles focus specifically on the experiences of LHS+ women, the small sample of literature allows us to provide a preview of what is being discussed in terms of the experiences of these populations. We must also point out the insufficiency and neglect that finding only 12 articles presents when it comes to understanding the needs of LHS+ women in medicine. This finding is even more prevalent for medical students and residents, given only five of the articles we identified discuss the experiences of these populations. After the brief overview, we discuss two themes we identified through our analysis.

### Overview of studies

For this section, we will provide a brief overview of the content found in all 12 articles identified in the critical narrative review process. The articles provided insights into the quantitative underrepresentation of LHS+ women in medicine and further explored some of their unique experiences within the field. Of the articles identified, 58% highlighted LHS+ women faculty and physicians. Consistently, 43% of those articles discussed LHS+ women faculty and physicians in terms of quantitative representation. Another 43% of articles discussed LHS+ women faculty and physicians’ experiences with discrimination. The final 14% of articles about LHS+ women faculty and physicians highlighted their knowledge about cardiovascular disease.

The remaining 42% of total articles were about LHS+ women medical students. Eighty percent of those articles highlighted LHS+ women medical students’ experiences with gender-based discrimination and depression. The final 20% of those articles were a perspective piece about the importance of mentorship programs for LHS+ women medical students.

### Key findings from studies

Although we found only 12 articles through our critical narrative review process, we felt it necessary to highlight key takeaways from the various research studies that could serve as resources for individuals working with LHS+ women in medicine. Representationally, LHS+ women are only 2.4% of the total US physicians and tend to be younger than other physicians, a fact we make clear at the onset of this paper [5]. Moreover, LHS+ women constitute 3.2% of medical school faculty and tend to be concentrated in the rank of assistant professor [30,31]. LHS+ women physicians or medical faculty often experience discrimination and have their qualifications and education questioned [32,33]. For LHS+ women physicians, researchers found that they tended to pick their specialty depending on which would provide them a more balanced lifestyle and most LHS+ women physicians did not take significant time off to care for their baby as new mothers [34]. Lastly, LHS+ women OBGYN physicians were found to be more knowledgeable about the risks of cardiovascular disease for women of the same ethnic group [35].

The remaining articles that explored the experiences of LHS+ women medical students detailed the Gender Based Discrimination (GBD) that students experience [36,37]. The women shared that they experienced GBD across multiple spaces on campus – at work, from professors, specialist medical doctors, and circulating nurses [36,37]. Furthermore, the women indicated that experiencing GBD impacted their career satisfaction, self-confidence, and sense of personal safety [36,37]. They felt that their gender greatly influenced their post-graduation career plans [37]. Research also highlighted that LHS+ women medical students were more likely to present symptoms of depression and anxiety compared to their male counterparts [38,39]. Despite this, they also have higher levels of empathy compared to their male counterparts [38].

### Invisibility and absence of LCFP

After reviewing all the articles identified through this critical narrative review, we have concluded two major considerations pertaining to research about LHS+ women across the medical continuum. Firstly, we found that LHS+ women were often made invisible in the medical education research reviewed for this study. Much of the research referenced LHS+ individuals, and while it can be assumed women were a part of those groups, one could not be sure unless LHS+ women are directly referenced. Disaggregating for gender is especially important because much of the literature was about the representation of LHS+ individuals in medical specialties and subspecialties. Further, several studies mentioned how both women and LHS+ individuals were underrepresented in several specialties. Thus, we argue the value of disaggregating data to understand the unique needs of all parties involved, specifically LHS+ women.

The second consideration is related to the absence of LCFP. Although we employed LCFP as an analytical tool, we were unable to find any evidence of decolonizing the research or recognition of LHS+ women who are physicians as whole beings in mind, body, and spirit. Conversely, we found the articles to promote Eurocentric values and research methods that placed LHS+ women at the margins, and not in ways promoted by LCFP. For example, rather than demystifying an academic medicine system that perpetuates an imbalance of mind, body, and spirit for LHS+ women, the authors of one article discussed the forced choices these women must make as recommendations to medical students. Additionally, instead of discussing the limitations of a system that forces LHS+ women into primary care and generalist specialties, the article highlighted these specialties as the best options for LHS+ women medical students who wanted children [34].

## Discussion and recommendations

Research confirms the importance of increasing diversity and fostering a sense of belonging for students of color [7,8,12]. However, in much of the medical education research students of color are referenced in terms of being members of a monolithic underrepresented in medicine (URIM) group rather than disaggregated for the various ethnic groups that exist. Consistently, medical education scholars have explored the needs of women in medicine [40,41], yet few scholars pay specific attention to the experiences of LHS+ women in medicine. To honor the unique racial, ethnic, and gender variations found among these learners, faculty, and physicians, researchers must take an anti-essentialism approach by disaggregating LHS+ women from URIM groups [6,42].

We underscore LCFP as a framework for exploring and more fully understanding the experiences of LHS+ women in medical education. This framework allows scholars to delve into and center on the unique experiences of LHS+ women in academia. As the LHS+ population in this country increases, LHS+ individuals in medicine are increasingly called on to serve as mediators and translators between the field of medicine and their communities [43]. LCFP allows for a deeper and more intentional analysis that recognizes how LHS+ women must balance living between the two worlds of their professional experiences within medicine while still being a present member for their family and community in their personal lives.

From the results of the critical narrative review, we see a disregard or erasure of LHS+ women in medical education literature. With the increasing number of LHS+ women entering medical school, scholars must contribute literature that recounts LHS+ women’s experiences to illuminate the successes medical education programs want to emulate. Furthermore, it is necessary to have more literature that centers on LHS+ women to highlight the practices that create challenges for these medical students [6]. Using LCFP as an analytical tool to address the dearth of literature on LHS+ women in medicine allows for scholars and medical educators to see and value the unique experiences LHS+ women offer and their contributions to the profession. LCFP can expose limitations of current practices and the lack of consideration for positive cross-cultural interactions, or the value LHS+ women contribute because of their ability to navigate multiple worlds. By using LCFP medical education researchers can explore experiences to eliminate the barriers that exist, thus fully supporting LHS+ women’s knowledge creation and identity development.

As medical educators and researchers continue developing programming for improving medical education students’ experiences, it is imperative to consider the multiple and intersectional identities of students, faculty, and physicians, as well as the perspectives that they bring. LCFP as an analytical lens de-centers Eurocentric values and honors multiple ways of being and knowing with a consideration of LHS+ women. This allows for varying points of entry for LHS+ women in medical education.

### Strengths and limitations

Upon reflection of this critical narrative review process, we want to share the strengths and limitations associated with this study. In terms of strengths, this critical narrative review was conducted by a group of interdisciplinary researchers who have expertise in the content area, thus allowing us to utilize a relevant theoretical framework for in-depth analysis. Because we were interested in the narratives about LHS+ women, the theoretical framework of LCFP and our world view supported an understanding for what was said and what was left unsaid. The study yielding 12 articles can be viewed as a limitation, but we also find that it justifies the necessity of research about the experiences of LHS+ women. Since this was a critical narrative review, and not a scoping or systematic review, we only needed to review one database to find manuscripts that met our inclusion criteria. Our inclusion criteria could also be seen as a limitation, as we only included articles published in America and written in English. Removing those criteria could have yielded more literature. However, our purpose was not only to find articles that discussed LHS+ women but to also find narratives about these women that could be useful in the development of recruitment and retention interventions. Lastly, the fact that we conducted this narrative review could be interpreted as a strength and limitation. As a strength, we had the opportunity to review the available literature again after major events took place in the United States to see if narratives about LHS+ women increased, which they did. As a limitation, there was a break in the consistency of the process given the two data sets were reviewed with such a large gap of time between them.

## Conclusion

This paper explored the narratives of LHS+ women available in medical education research. The lack of representation for LHS+ women in medical education literature ultimately points to the need for more intentional exploration of this group of women’s experiences as medical students, residents, faculty, and practicing physicians. Furthermore, without the presence of LHS+ women in medical education research, there is a narrow opportunity to explore what their medical school experiences are and have been to tailor positive interventions. This limits how medical school educators should address the possibilities of enhancing or replicating those experiences and environments in medical schools and for LHS+ women.

## Previous presentations

The findings from this project were presented at the American Educational Research Association (AERA) conference, which convened in April 2023.
